# Cohort profile: why do people keep hurting their back?

**DOI:** 10.1186/s13104-020-05356-z

**Published:** 2020-11-17

**Authors:** David M. Klyne, Wolbert van den Hoorn, Mary F. Barbe, Jacek Cholewicki, Leanne M. Hall, Asaduzzaman Khan, Roberto Meroni, G. Lorimer Moseley, Michael Nicholas, Lee O’Sullivan, Rachel Park, Glen Russell, Michele Sterling, Paul W. Hodges

**Affiliations:** 1 NHMRC Centre of Clinical Research Excellence in Spinal Pain, Injury and Health, School of Health and Rehabilitation Sciences, The University of Queensland, Brisbane, QLD 4072 Australia; 2 Department of Anatomy and Cell Biology, Lewis Katz School of Medicine, Temple University, Philadelphia, USA; 3 MSU Center for Orthopedic Research, Department of Osteopathic Surgical Specialties, Michigan State University, East Lansing, MI USA; 4 School of Health and Rehabilitation Sciences, The University of Queensland, Brisbane, Australia; 5 Department of Physiotherapy, LUNEX International University of Health, Exercise and Sports, Differdange, Luxembourg; 6 IIMPACT, University of South Australia, Adelaide, Australia; 7 Pain Management Research Institute, Royal North Shore Hospital, The University of Sydney, Sydney, Australia; 8 NHMRC Centre of Research Excellence in Road Traffic Injury Recovery, The University of Queensland, Brisbane, Australia; 9 Recover Injury Research Centre, The University of Queensland, Brisbane, Australia

**Keywords:** Low back pain (LBP), Acute to chronic, Biological factors, Psychological factors, Social factors, Behavioural factors

## Abstract

**Objective:**

Low back pain (LBP) is one of the most disabling and costly conditions worldwide. It remains unclear why many individuals experience persistent and recurrent symptoms after an acute episode whereas others do not. A longitudinal cohort study was established to address this problem. We aimed to; (1) evaluate whether promising and potentially modifiable biological, psychological, social and behavioural factors, along with their possible interactions, predict LBP outcome after an acute episode; (2) compare these factors between individuals with and without acute LBP; and (3) evaluate the time-course of changes in these factors from LBP onset. This paper outlines the methodology and compares baseline characteristics between acute LBP and control, and LBP participants with and without follow-up.

**Results:**

133 individuals with acute LBP and 74 pain-free individuals participated. Bio-psycho-social and behavioural measures were collected at baseline and 3-monthly for 12 months (LBP) or 3 months (control). Pain and disability were recorded fortnightly. Baseline characteristics were mostly similar between those who did and did not return for follow-up. Initial analyses of this cohort have revealed important insights into the pathways involved in acute-to-chronic LBP. These and future findings will provide new targets for treatment and prevention of persistent and recurrent LBP.

## Introduction

Low back pain (LBP) is the world’s leading cause of disability [1] and is associated with enormous and escalating costs to society [2]. Most of this burden is attributed to the condition when LBP becomes persistent or recurrent. Why some individuals with acute LBP recover, whereas others do not [3, 4], is largely unknown.

Although psychosocial factors have generally been considered stronger predictors of long-term outcome than diagnostic or injury-related factors [5, 6], they only explain a small proportion of the variance in outcome [6, 7]. Biological factors have largely been dismissed and the few that have been comprehensively addressed (e.g., muscle strength/endurance [8]) have little relation to outcome [9]. With this incomplete understanding of factors related to LBP outcome, it is not surprising that most treatments have modest effects at best [10] and are generally unable to prevent recurrence/persistence of pain [11].

We argue that three issues underlie a fresh approach to this problem. First, novel biological factors that could plausibly contribute to LBP outcome have been identified in cross-sectional studies, e.g., systemic inflammation, trunk muscle morphology/function, and processing of pain. Up until now, these potentially modifiable factors had not been tested longitudinally from the initial onset of symptoms, and analyses of the early time-points from the cohort presented in this paper are providing promising results [12–14]. Second, although interaction between biological, psychological and social factors is implied in the biopsychosocial model of pain, this interaction has received little attention in past longitudinal studies of LBP outcome. Again, early analyses from the present cohort have revealed interaction between features such as depression, cytokines and poor LBP outcome [12–16]. Third, there is growing evidence that behavioural factors such as sleep interact with the “biopsychosocial” components of LBP, but their contribution to outcome is unknown. This too has been supported by early observations [12, 14]. Further, little is known of the time course of changes in each of the biopsychosocial domains over 12 months following an acute LBP episode. There is strong foundation to evaluate whether candidate biological factors, along with their possible interaction with psychosocial factors, contribute to the transition from acute LBP to that of persistent/recurrent symptoms.

The purpose of this paper is to provide a profile of a cohort study that aims to; (1) evaluate whether outcome after an acute episode of LBP can be predicted by the most promising bio-psycho-social factors and/or the interactions between them; (2) compare these factors between individuals with and without acute LBP; and (3) evaluate the time course of changes in these factors following LBP onset. This paper outlines the participants, measures and data collection schedule, and compares baseline characteristics between acute LBP and control, and follow-up and non-follow-up LBP participants.

## Main text

### Methods

#### Study design

This longitudinal cohort study involved measures of variables within the biological, psychological and social domains (Table 1) at multiple time-points for 12 months (Additional file 1: Table S1). Measures of sleep, physical activity, alcohol consumption and smoking were grouped separately in a “behavioural” domain as they cross between classical domains. Eligible participants completed a series of detailed online questionnaires related to their pain and disability level, health, demographics, behaviour, and psychosocial status within 24 h of undertaking a laboratory-based session (~ 4 h) at the University of Queensland to assess biological variables. Measures (laboratory-based biological measures and online questionnaires) were repeated at 3, 6 and 9 months for LBP participants, and at 3 months for control participants. At 12 months, questionnaires were completed by all participants in the LBP group in addition to a separate 12-month recall questionnaire relating to the trajectory of their LBP since initial assessment for the study. Participants were also instructed (and reminded) via email to report their pain and disability level every fortnight for 3 (controls) or 12 months (LBP) via an online survey. For some analyses that have been conducted to date, these pain and disability data have been used to classify LBP participants as either “unrecovered”, “partially recovered” or “recovered” at follow-up (for details see Additional file 1: Table S2).

**Table 1:** Detailed description of measures

Measure	Description	Units/range
*Demographic, health & function*		
Age, height, weight, sex	Self-reported age, height, weight and sex	Years, cm, kg, male/female
BMI	Weight (kg) divided by the squared height (cm)	Numerical
Co-morbidities	Self-selected disease(s)/condition(s) other than LBP from a list (including “other”)	Yes/no, type
Previous LBP	Self-reported previous incidence(s) of LBP not including the current (study entry) episode	Yes/no
Health care/medication usage	Self-reported health care and medication frequency of use and type for LBP	Yes/no, frequency, type
Low-Back Outcome Scale (LBOS [17], *measured in LBP only*)	Questionnaire: evaluates pain and physical function. Consists of 13 differently weighted items that assess current pain, function (e.g., employment, domestic chores and sport activities), and the frequency of use of medical treatments/consultations and analgesics with respect to the respondent’s LBP	0–75: ↑score = ↑function, 0–29 = poor, 30–49 = fair, 50–64 = good, ≥ 65 = excellent
*Psychological*		
Centre for Epidemiological Studies of Depression Scale (CES-D [18])	Questionnaire: evaluates depressive symptoms. Consists of 20 items. Respondents’ rate how often over the past week they experienced symptoms associated with depression using a four-point Likert scale ranging from 0 (“rarely or none of the time”) to 3 (“most or all of the time”)	0–60: ↑score = ↑depressive symptoms, > 15 = clinically significant depressive symptoms
Pain catastrophizing scale (PCS [19])	Questionnaire: evaluates thoughts and feelings related to pain suggestive of catastrophic cognitions. Consists of 13 items. Responses to questions are quantified on a five-point Likert scale ranging from 0 (“not at all”) to 4 (“all the time”) with respect to how often the respondent experiences certain thoughts and feelings when in pain. Yields a total score as well as three subscale scores of magnification (“I become afraid that the pain will get worse”: 3 items), rumination (“I worry all the time whether the pain will end”: 4 items) and helplessness (“I feel I can't go on”: 6 items)	0–52: ↑score = ↑pain catastrophizing
		Subscales: magnification (0–12), rumination (0–16) helplessness (0–24)
Fear-Avoidance Beliefs Questionnaire (FABQ [20], *measured in LBP only*)	Questionnaire: evaluates fearful and avoidant behaviours. Consists of 16 items in which participants’ rate their agreement with each statement on a seven-point Likert scale ranging from 0 (“completely disagree”) to 7 (“completely agree”). Two subscales measure the agreement of statements related to physical activity (FABQ-PA: 4 items) and work (FABQ-W: 7 items)	0–96: ↑score = fear-avoidance beliefs
		Subscales: FABQ-PA (0–24), FABQ-W (0–42)
Pain Self-Efficacy Questionnaire (PSEQ [21], *measured in LBP only*)	Questionnaire: evaluates the confidence individuals have in performing activities while in pain. Consists of 10 items. Respondents’ rate how confidently they can perform a range of activities using a seven-point Likert scale	0–60: ↑score = ↑self-efficacy beliefs
*Social*		
Marital status	Self-selected marital status (e.g., never/currently married, separated, cohabitating, etc.) from a list	Type
Education level	Self-selected education level (e.g., school certificate, bachelor/postgraduate degree, etc.) from a list	Type
Employment status	Self-selected employment status (e.g., full-time/full duties, part-time, unemployed, etc.) from a list	Type
Type of work	Self-selected primary occupation (e.g., professional, technician, clerk, etc.) in the last 12 months from a list	Type
Job satisfaction	Self-reported job satisfaction using a seven-point NRS ranging from “extremely dissatisfied” to “extremely satisfied”	0–6: ↑score = ↑job satisfaction
Job Content Questionnaire (JCQ [22])	Questionnaire: evaluates psychosocial demands resulting from the respondent’s job. Consists of 27 items re-grouped into several dimensions. Responses to each item are quantified on a four-point Likert scale from 1 (“totally disagree”) to 4 (“totally agree”)	Job skill discretion (12–48, ↑score = ↑discretion), job decision-making authority (12–48, ↑score = ↑authority), job demands (12–48, ↑score = ↑demands), job decision latitude (24–96, ↑score = ↑latitude), co-worker support (4–16, ↑score = ↑support), supervisor support (4–16, ↑score = ↑support), job insecurity (3–12, ↑score = ↑insecurity)
Sick days over last 12 months	Self-reported number of sick-days taken from work over the previous 12 months	Numerical
Reason(s) for not working	Self-selected reason(s) for not working for pay (e.g., caring for family, studies/training, ill health, etc.) from a list	Yes/no, type
Sickness benefits	Self-reported sickness benefits associated or not associated with the participants’ LBP	Yes/no, for LBP/other
Impending compensation	Self-reported impending compensation associated with the participants’ LBP	Yes/no
*Biological*		
Systemic inflammation	Laboratory measure: Serum concentrations of TNF, IL-6, IL-1β and CRP. Venous blood was drawn, clotted (30 min, room temperature), and serum was separated by centrifugation (2500 rpm, 15 min) before storing at − 80 °C. Concentrations of each biomarker were determined in duplicate using “high sensitive” (assay sensitivity: CRP = 0.022 ng/ml, IL-6 = 0.110 pg/ml, IL-1β = 0.14 pg/ml, TNF = 0.191 pg/ml) enzyme-linked immunosorbent assays (ELISA, R&D Systems, Minneapolis, MN). Zero was allocated for values below the reported sensitivity of the test [14, 16]	TNF (pg/ml), IL-6 (pg/ml), IL-1β (pg/ml), CRP (ng/ml)
Pain processing	Laboratory measure: Pain thresholds to pressure (PPT), heat (HPT) and cold (CPT) were assessed at the back (LBP – site of most pain on palpation; control – fixed site ~ 5 cm rostral [toward the head] and lateral to the center of the lumbo-sacral junction divided randomly between the left and right side) and either the thumb nail bed (PPT) or proximal volar aspect of the forearm (HPT and CPT) [12, 15]. CPM was assessed based on our previous work [23] that validated the use of PPT as a test stimulus (TS) and noxious contact heat as the conditioning stimulus (CS). TS and CS were applied to the lower back or forearm. CPM was measured on three occasions (separated by a 15-min break) using different anatomical locations and stimuli (TS/CS) arrangements as reported previously [12, 15]. The CPM response was calculated as the difference between the TS scores obtained before and during the CS. A higher TS score during the CS than baseline indicated pain inhibition (expressed as a positive value). A lower TS score during the CS than baseline indicated pain facilitation (negative value)	PPT (kPa, ↑score = ↑pain threshold), HPT (°C, ↑score = ↑pain threshold), CPT (°C, ↑score = ↓pain threshold), CPM (kPa, > 0 = pain inhibition, < 0 = pain facilitation)
Multifidus muscle morphology	Laboratory measure: Multifidus muscle cross sectional area was measured at the level of each spinous process between the first lumbar (L1) and first sacral (S1) vertebra on both sides of the body (totalling 12 images) using a high resolution ultrasound system (LOGIC 9; GE Company, Milwaukee, WI), with a linear array 10 MHz transducer [24]	Cross-sectional area (cm^2^)
Trunk muscle coordination	Laboratory measure: Latency of response of superficial trunk muscle activity to unloading was assessed using an established paradigm [25]. Participants sat in a semi-sitting position with their pelvis fixed and a cable attached either behind or in front of their trunk via a harness. On instruction, participants pulled against the cable with a force of 12.5% of their body weight using visual feedback (target on a computer screen). The cable was released at an unpredictable time for 10 repetitions in each direction and the response of 12 abdominal and back muscles were recorded with surface EMG electrodes using placement described previously	Muscle activity (EMG)
Trunk mechanical properties	Laboratory measure: Effective trunk stiffness, mass and damping was estimated following trunk perturbation with the trunk modeled as a linear second-order system [26]. Participants sat in a frame with equal weights (7.5% body weight) attached to the front and back of the trunk via pulleys such that the masses were balanced and the trunk could move freely with minimal muscle activity. The trunk was perturbed by the unexpected release of one of the weights. The task was repeated 10 times in each direction with the order of directions randomised. Trunk kinematics and cable force were used to estimate system properties	Mass (kg), stiffness (N/m), damping (Ns/m)
Trunk postural control	Laboratory measure: Dynamic trunk control was assessed with participants balancing on a seat with a curved base placed on a force plate to record centre of pressure (CoP) [27]. Participants performed three 30-s trials (separated by a 1 min rest period) with eyes open, eyes closed and with feedback of the seat position in the anteroposterior direction (target on a computer screen). In an additional trial, participants were perturbed by release, at an unexpected time, of a weight (3% body weight) attached behind the trunk. Feedback was provided until the perturbation. Participants were instructed to regain balance as fast as possible three times under four conditions: eyes open, eyes closed, feedback (as above), and feedback up until perturbation induced by release of the weight. Coordinates of CoP were recorded as well as trunk muscle activity (as for trunk muscle coordination) and kinematics using a motion capture system	Balance (CoP), muscle activity (EMG), kinematics (degrees)
Standing postural control	Laboratory measure: Postural control was measured with participants standing barefoot and blindfolded on a force plate for 75 s. To test the effect of disruption of proprioception at the calf and lower back, the task was repeated with vibrators (~ 60 Hz, 1 mm amplitude) attached bilaterally over the Achilles tendon and lumbar paraspinal muscles [28], separately, in random order. Vibrators were switched on for 15 s at ~ 15 s after the start of the trial. Coordinates of CoP were recorded	Balance (CoP)
Lumbopelvic motion	Laboratory measure: Angular measures of limb movement and lumbopelvic motion were calculated across time during active/passive knee flexion and active/passive hip rotation (both lateral and medial) in prone using a motion capture system [29]	Kinematics (degrees)
Lumbopelvic control during gait	Laboratory measure: Lumbopelvic/trunk kinematics and trunk muscle activity (EMG) was assessed during 3 min of treadmill walking at 3 km/h and 5 km/h [30, 31]	Muscle activity (EMG), kinematics (degrees)
*Behavioural*		
Pittsburgh Sleep Quality Index (PSQI [32])	Questionnaire: evaluates sleep duration and quality. Consists of 19 items that cover seven dimensions, including subjective sleep quality, sleep duration and latency (time it takes to fall asleep), and the frequency and severity of specific sleep-related complaints in the previous month. Scores from each dimension (range: 0–3) are individually reported as component scores and summed to derive a sleep quality maximum score	0–21: ↑score = ↓sleep quality, > 5 = poor sleeper
		Component scores (all 0–3): duration of sleep, sleep disturbance, sleep latency, day dysfunction due to sleepiness, sleep efficacy, overall sleep quality, sleep medications
		Sleep hours per night (h)
International Physical Activity Questionnaire (IPAQ [33])	Questionnaire: evaluates health-related physical activity. Consists of seven items that assess four domains of physical activity over the previous week, including vigorous activity (activities that make breathing much harder than normal), moderate activity (activities that make breathing somewhat harder than normal), walking and time spent sitting	↑score = ↑physical activity (refer to scoring manual for calculating and interpreting MET scores and activity categories)
Alcohol Use Disorders Identification Test (AUDIT [34])	Questionnaire: evaluates alcohol consumption, dependence and drinking-related problems. Consists of 8 items (i.e., shortened version of the full 10-item AUDIT) that address four areas: alcohol consumption (quantity and frequency), drinking behaviour and dependence, alcohol related psychological effects and alcohol related problems. Responses to each item are quantified on a five-point Likert scale from 0 to 4	0–32: ↑score = ↑level of alcohol problem
Past/current smoking status	Self-reported past and current smoking history	Yes/no, duration, quantity
*Fortnightly (for 12 months)*		
Pain	Self-reported pain intensity over the last week using an 11-point NRS ranging from “none” to “worst imaginable”	0–10: ↑score = ↑pain
Roland Morris Disability Questionnaire (RMDQ [35])	Questionnaire: evaluates disability caused by LBP. Involves 28 items (i.e., extended version of the standard 24-item RMDQ) associated with physical functions likely to be affected by LBP. An item receives a score of 1 if it is applicable to the respondent or a score of 0 if it is not	0–28: ↑score = ↑disability
12-month LBP trajectory	Questionnaire: evaluates the trajectory of LBP symptoms over 12 months from study commencement. Consists of a series of questions (asked at 12 months) that address how the respondent’s current LBP compares with their LBP at the start of the study, the frequency and duration at which the responder experienced periods without pain, periods of recurrence/persistence, and/or periods of markedly worse symptoms, based on their 12-month recall. In addition, respondents are asked to classify their LBP experience into one of seven trajectories using visual and word descriptions	Yes/no, duration, frequency, trajectory type

*TNF* tumor necrosis factor, *IL-6* interleukin-6, *IL-1β* interleukin-1β, *CRP* C-reactive protein, *PPT* pressure pain threshold, *CPT* cold pain threshold, *HPT* heat pain threshold, *CPM* conditioned pain modulation, *TS* test stimulus, *CS* conditioning stimulus, *EMG* electromyography, *CoP* centre of pressure

#### Participant recruitment

**Fig. 1:**
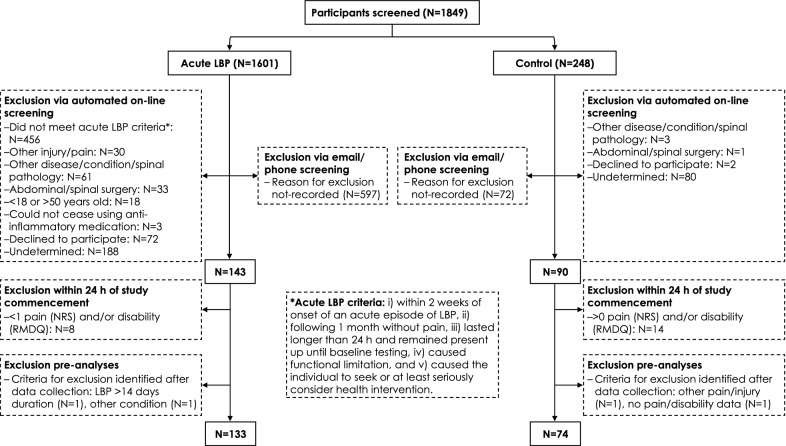
Cohort flow diagram

A total of 1849 individuals from Brisbane (and surrounds), Australia, were screened between April 2012 and September 2017 (Fig. 1). Participants were recruited through advertisements around the University campus and local community, social media, three nearby hospitals and via a professional recruitment agency (Trialfacts).

Screening was conducted using two different methods. Initially, eligibility was determined via email and/or phone, and when this method was used the reason(s) for exclusion at this initial screening were not recorded. This was replaced with an automated online screening questionnaire from April 2014, with reasons for exclusion recorded. The inclusion and exclusion criteria for LBP participants are outlined in Additional file 1: Table S3. Participants did not need to be experiencing their first ever LBP episode. Previous LBP was recorded for inclusion as a covariate. Control participants were included if they had not experienced LBP within the last month in addition to meeting the exclusion criteria in Additional file 1: Table S3.

At the first laboratory testing session (after initial screening), eligibility for inclusion were confirmed using data from the baseline questionnaire (completed within 24 h of the first laboratory-based session), to ensure that the participant’s average level of pain and LBP-related disability in the past week exceeded the inclusion threshold (≥ 1/10 for pain; ≥ 1/24 for disability). Potential control participants who reported a score > 0 on a 0 (“no pain”) to 10 (“worst pain imaginable”) numerical rating scale (NRS) and/or the Roland Morris Disability Questionnaire (RMDQ [35], [for definition of measures see Table 1]), or provided no scores were excluded from the study (N = 14). Potential LBP participants who reported < 1 on the pain NRS and/or the RMDQ, or provided no scores in the past week were excluded from the study (N = 8). After data collection, criteria for exclusion were identified for two participants with LBP (multiple sclerosis [N = 1], duration of LBP > 14 days [N = 1]) and two control participants (no pain/disability data [N = 1], pain/injury in another body region [N = 1]). These participants were excluded from the dataset. The final cohort included 133 and 74 participants in the LBP and control groups, respectively, for analyses.

#### Measurements

Details of the measures and at what time-point(s) they were implemented are presented in Table 1 and Additional file 1: Table S1. All variables were measured in a standardised order for each participant. Biological measures were those we considered to be the most promising candidate factors for predicting LBP recurrence/persistence based on previous research and plausible rationales founded on clinical, epidemiological and fundamental research. For psychological measures, we considered three key domains of relevance in LBP: cognitive (expectations, beliefs, and perceptions concerning pain) [6, 36–38], emotional (distress, anxiety, and depression) [5], and behavioural (coping, pain behaviour, and activity/activity avoidance) [6, 36, 37]. Social measures were selected based on the Multinational Musculoskeletal Inception Cohort Study (MMICS) guidelines [39]. These guidelines were developed by an international expert team with review of the best available evidence from systematic/narrative reviews and expert consensus. As it was not our intention to withhold treatment over the study period, we collected information regarding health care and medication use so that treatment variables can be included as covariates. The total number of variables was restricted to limit the required participant sample size, minimise the potential for over-fitting, and for cost–benefit.

#### Sample size

A sample size of 217 was calculated based on power to detect predictor variables using complex multiple regression models (i.e., 28 predictor variables, 5 a priori selected interactions) and growth curve modelling methods, while allowing for loss to follow-up. The planned sample size was not achieved due to feasibility issues as outlined in the “limitations” section. Although the achieved sample size (N = 133) limits the ability to examine numerous interactions simultaneously, reduced model sizes and alternative methods (e.g., cluster analysis) have been applied successfully on data from this cohort [12–16].

#### Data analysis

All questionnaire-based measures (that could be quantitatively analysed) at baseline were compared between: (1) LBP and control participants, and (2) LBP participants who did and did not follow-up at 3, 6, 9 and 12 months.

### Results

#### Participant characteristics at baseline

The characteristics of the study participants are described in Additional file 1: Table S4. Compared to controls, LBP participants were/had: taller and heavier, a higher BMI, a higher prevalence of comorbidities, a higher incidence of previous LBP, higher depressive and pain catastrophizing symptoms, higher self-reported job demands, more sick days (over the last 12 months), poorer sleep quality, more likely to have a history of cigarette smoking, and more likely to have performed vigorous physical activity on less days in the previous week.

#### Participant attrition

Of the 133 eligible acute LBP participants who were enrolled in the study and provided baseline data, 35 (26%) were lost to follow-up for their laboratory-based measures (i.e., did not attempt/complete any biological measures) at 3 months, a further 9 at 6 months (total lost to follow-up = 44, 33%), and a further 5 at 9 months (total lost to follow-up = 49, 37%). As biological measures were not performed at 12 months, all participants were invited to complete the standard 3-montly questionnaire in addition to a separate recall questionnaire at 12 months, irrespective of whether or not they had continued or dropped out earlier. One or both of these were fully/partly completed by all but 41 of the 133 LBP participants who started the study (follow-up at 12 months: N = 92). For participants that did follow-up, Additional file 1: Table S5 shows the number of those that provided valid data for each of the 3-monthly questionnaire-based measures at each respective time-point. Ten control participants did not return for follow-up at 3 months. With respect to the completion rate of fortnightly pain (NRS) and disability (RMDQ) questionnaires, 85% (1505 of 1770) were completed by LBP participants who were retained for follow-up (i.e., up to 3, 6, 9 or 12 months) within 7 days of each questionnaire being issued, and 91% (282 of 310) were completed by control participants (i.e., up to 3 months).

#### Comparison of follow-up and non-follow-up participants

Comparison of baseline characteristics between LBP participants who did and did not follow-up for laboratory-based measures at 3, 6 and 9 months, and questionnaire measures at 12 months, revealed some differences, as shown in Table 2.

**Table 2:** Comparison of baseline characteristics between participants with LBP who did (FU) and did not (NFU) follow-up at 3, 6, 9 and 12 months

Characteristic	3 months			6 months			9 months			12 months		
	Summary statistics		P-value	Summary statistics		P-value	Summary statistics		P-value	Summary statistics		P-value
	FU (N = 98)	NFU (N = 35)		FU (N = 89)	NFU (N = 44)		FU (N = 84)	NFU (N = 49)		FU (N = 92)	NFU (N = 41)	
*Demographic, health & function*												
Age (years)^‡^	28 (22–34)	24 (20–32)	0.074	27 (22–34)	26.5 (20.5–33.5)	0.362	27.5 (22.5–34)	25 (21–34)	0.264	27 (22.5–35)	25 (20–31)	0.090
Sex (% female)	48.0	62.9	0.130	49.4	56.8	0.423	50.0	55.1	0.570	51.1	53.7	0.784
Height (m)^†*^	1.73 (0.09)	1.70 (0.09)	0.078	1.73 (0.09)	1.71 (0.09)	0.173	1.73 (0.09)	1.72 (0.09)	0.615	1.73 (0.09)	1.71 (0.09)	0.507
Weight (kg)^‡*^	73 (63–82)	72 (56–85)	0.746	73 (62–82)	73.5 (60.5–84.5)	0.815	72.5 (62.5–82.5)	74 (61–84)	0.744	71.5 (60.5–81.5)	75 (62–85)	0.680
BMI (kg/m^2^)^‡^	24.0 (21.4–26.3)	25.2 (21.0–27.7)	0.409	24.0 (21.4–26.3)	24.1 (21.7–27.5)	0.449	24.0 (21.8–26.3)	23.9 (21.1–27.2)	0.961	23.9 (21.4–26.1)	25.9 (21.2–27.4)	0.313
Comorbidity (yes, %)	41.8	54.3	0.204	42.7	50.0	0.426	40.5	53.1	0.159	0.5	41.5	0.572
Previous LBP (yes, %)	92.9	88.6	0.429	92.1	90.9	0.809	91.7	91.8	0.973	92.4	90.2	0.678
Healthcare utilization (yes, %)	18.4	23.5	0.514	20.2	18.6	0.826	21.4	16.7	0.508	19.6	20.0	0.954
Medication utilization (yes, %)	18.8	32.1	0.152	21.1	25.0	0.650	19.7	26.8	0.390	16.7	34.3	*0.040*
Function (LBOS)^†^	48.4 (11.2)	42.7 (10.8)	*0.012*	48.3 (10.7)	44.1 (12.2)	*0.048*	48.2 (10.5)	44.6 (12.4)	0.077	48.1 (11.0)	44.1 (11.7)	0.060
*Outcome*												
Pain (NRS)^‡^	5 (3–6)	6 (5–7)	*0.019*	5 (3–6)	6 (4–7)	0.078	5 (3–7)	5 (4–7)	0.506	5 (3–6.5)	6 (4–7)	0.147
Disability (RMDQ)^‡^	6 (3–9)	7 (4–11)	0.140	6 (3–9)	6.5 (4–10.5)	0.293	6 (4–9)	6 (4–10)	0.613	6 (4–9)	7 (4–10)	0.257
*Psychological*												
Depressive symptoms (CES-D)^‡^	11 (7–17)	16 (10–25)	*0.003*	10 (7–17)	16 (10–22)	*0.008*	10 (7–18)	15 (10–22)	*0.004*	10 (7–18)	15 (11–23)	*0.017*
Pain catastrophizing (PCS)^‡^	10 (6–16)	19 (7–26)	0.057	10 (6–16)	13 (7–26)	0.125	10 (6–16)	14 (7–26)	0.106	10 (6–16)	17 (9–26)	*0.019*
Fear avoid.-work (FABQ-W)^‡^	11 (2–17)	15 (6–22)	0.136	12 (2–17)	12 (3–24)	0.223	12 (3–17)	13 (4–23)	0.144	12 (4–17)	10 (0–23)	0.960
Fear avoid.-activity (FABQ-PA)^†^	14.7 (5.5)	14.9 (5.4)	0.835	14.7 (5.3)	14.8 (5.8)	0.944	14.6 (5.6)	15.0 (5.4)	0.686	14.8 (5.4)	14.7 (5.8)	0.926
Pain self-efficacy (PSEQ)^‡^	48 (40–53)	41 (32–48)	*0.008*	48 (40–52)	44 (32–48)	*0.021*	48 (40–53)	44 (32–48)	*0.009*	47 (40–53)	45 (32–48)	*0.047*
*Social*												
Marital status (not married/cohabitating, %)	65.3	58.8	0.498	64.0	62.8	0.888	64.3	62.5	0.837	63.0	65.0	0.830
Edu. level (secondary school/below, %)	24.5	26.5	0.818	24.7	25.6	0.915	23.8	27.1	0.676	25.0	25.0	1.000
Empl. status (unemployed, %)	25.5	14.7	0.195	25.8	16.3	0.219	25.0	18.8	0.410	20.7	27.5	0.388
Job satisfaction (NRS)^†^	4.3 (1.2)	3.8 (1.1)	0.070	4.3 (1.2)	3.9 (1.2)	0.092	4.3 (1.2)	3.8 (1.1)	*0.025*	4.3 (1.2)	3.8 (1.1)	*0.044*
Job skill discretion (JCQ)^‡^	38 (32–42)	34 (28–40)	0.249	38 (32–42)	34 (30–40)	0.213	38 (32–42)	34 (30–39)	0.148	38 (32–41)	36 (32–40)	0.470
Job decision-making authority (JCQ)^‡^	40 (32–44)	36 (28–42)	0.081	40 (32–44)	36 (28–44)	0.136	40 (36–44)	36 (28–40)	*0.026*	40 (32–44)	36 (28–44)	0.254
Job demands (JCQ)^‡^	32 (27–36)	33 (31–38)	0.096	32 (27–36)	33 (30–38)	0.068	32 (27–36)	33 (29–38)	0.214	32 (29–36)	33 (26–38)	0.922
Job decision latitude (JCQ)^‡^	76 (66–86)	70 (56–80)	0.152	76 (67–86)	70 (62–82)	0.172	77 (68–86)	70 (60–80)	0.058	76 (66–86)	74 (62–82)	0.353
Co-worker support (JCQ)^‡^	12 (12–14)	12 (11–12)	* < 0.001*	12 (12–14)	12 (11–12)	*0.002*	13 (12–14)	12 (11–12)	* < 0.001*	12 (12–14)	12 (12–13)	0.190
Supervisor support (JCQ)^‡^	12 (12–15)	12 (11–14)	0.341	12 (12–15)	12 (11–14)	0.411	12 (12–16)	12 (11–13)	0.122	12 (11–16)	12 (11–13)	0.530
Job insecurity (JCQ)^‡∆^	5 (4–7)	6 (5–8)	0.156	5 (4–7)	6 (5–7)	0.088	5 (4–7)	6 (5–8)	*0.046*	5 (4–7)	5 (5–7)	0.308
Sick days over last 12 months^‡*^	2 (0–4)	2 (0–5)	0.618	2 (0–4)	2 (0–5)	0.711	2 (0–4)	2 (0–5)	0.803	2 (0–4)	2 (0–5)	0.975
Sickness benefits for LBP (yes, %)	1.3	3.6	0.439	1.4	2.8	0.621	1.5	2.4	0.732	0.0	5.7	*0.041*
Impending compensation (yes, %)	6.3	17.9	0.072	7.0	13.9	0.250	7.6	12.2	0.425	4.2	20.0	*0.008*
*Behavioural*												
Sleep quality (PSQI)^†^	9.0 (3.3)	10.0 (3.2)	0.215	9.0 (3.4)	9.7 (3.1)	0.337	9.0 (3.5)	9.6 (3.0)	0.334	9.2 (3.5)	9.1 (2.9)	0.828
Alcohol use/related problems (AUDIT)^‡^	3 (2–6)	3 (1–7)	0.996	3 (2–6)	3 (1–7)	0.778	3 (1–6)	3 (2–8)	0.617	3 (2–6)	3 (1–8)	0.742
Previous/current smoker (yes, %)	35.7	38.2	0.792	36.0	37.2	0.888	34.5	39.6	0.561	35.9	37.5	0.858
Current smoker (yes, %)	6.1	11.8	0.284	6.7	9.3	0.602	6.0	10.4	0.351	5.4	12.5	0.159
Vig. phys. activity days/week (IPAQ)^‡*^	2 (0–3)	1 (0–3)	0.783	2 (0–3)	2 (0–4)	0.312	2 (0–3)	2 (0–3)	0.606	2 (0–3)	1 (0–2)	0.233
Vig. phys. activity time/day (min, IPAQ)^‡*^	20 (0–60)	20 (0–60)	0.728	18 (0–60)	30 (0–60)	0.263	20 (0–60)	25 (0–60)	0.700	28 (0–60)	0 (0–60)	0.575
Mod. phys. activity days/week (IPAQ)^‡*^	2 (1–4)	2 (0–4)	0.979	2 (1–4)	2 (0–4)	0.743	2 (1–4)	2 (0–4)	0.874	2 (1–4)	2 (0–4)	0.228
Mod. phys. activity time/day (min, IPAQ)^‡*^	30 (0–60)	30 (0–30)	0.082	30 (0–60)	23 (0–30)	0.070	30 (0–60)	30 (0–35)	0.113	30 (0–60)	30 (0–45)	0.233
Days/week walking for ≥ 10 min (IPAQ)^‡*^	6 (4–7)	6 (4–7)	0.806	6 (4–7)	6 (3–7)	0.594	6 (4–7)	6 (4–7)	0.747	6 (4–7)	6 (4–7)	0.729
Walking time/day (min, IPAQ)^‡*^	30 (18–60)	30 (20–90)	0.158	30 (20–45)	35 (20–75)	0.118	25 (15–40)	45 (20–75)	*0.023*	25 (20–45)	48 (20–60)	0.076
Sitting time/day (min, IPAQ)^†*^	430.6 (174.8)	385.0 (211.3)	0.298	429.4 (173.6)	394.3 (209.9)	0.401	437.3 (176.1)	381.3 (198.6)	0.166	428.7 (173.6)	393.5 (212.5)	0.410

Baseline variable (characteristic) summary statistics (mean [SD]^†^, median [IQR]^‡^ or percentage) compared between low back pain (LBP) participants who did (FU) and did not follow-up (NFU), separately at 3, 6, 9 (for laboratory-based measures) and 12 (questionnaire measures) month time-points using *t* tests (continuous data, normally distributed), Mann–Whitney *U* tests (continuous data, not normally distributed) or Chi squared tests (categorical data). *Edu*. education, *Empl* employment, *Vig* vigorous, *Mod* moderate, *min* minute. Refer to Table 1 for other abbreviations.

*If a participant provided a range of values in response to a question (e.g., 30–60 min), the average was calculated for analysis purposes.

^∆^Participants who answered “other” to *question 25* of the Job Content Questionnaire were removed prior to analysing the “job insecurity” scale.

### Discussion

This paper profiles the only acute LBP cohort in which detailed biological, psychological, social and behavioural factors have been longitudinally and frequently collected, to date. The cohort has great potential to provide unique insight into the features that may predict and/or mediate long-term outcome [40].

The findings of baseline (acute LBP) characteristics presented here provide a foundation for future longitudinal analyses. Whether the findings can be generalised to a larger or clinical sample of individuals with early-acute LBP requires further and detailed studies of the condition during the early-acute phase. Despite the rate of loss to follow-up, most occurred after the first session, and baseline characteristics were generally similar between those who did and not return for follow-up.

Initial analyses of this cohort have revealed specific immune and nervous system features associated with the transition to persistent/recurrent LBP, and that various psychological and behavioural factors shape these relationships [12–16]. Ongoing analyses focus on elucidating the role of trunk neuromuscular, kinematic, mechanical and morphological properties, along with their possible interactions with psychosocial/behavioural features, in predicting LBP outcome.

## Limitations

- The strict “acute LBP” inclusion criteria (i.e., within 2 weeks of onset of a LBP episode following 1 month without pain) rendered recruitment challenging – > 50% of screened individuals did not meet these criteria.
- Study measures and follow-up procedures imposed substantial burden and explains the reported attrition.
- Missing data due to attrition was high as is usual in longitudinal cohorts, and statistical approaches (e.g., mixed effects models) have been, and will continue to be, used to minimise bias.
- The smaller than expected sample size limits the types of analyses to investigate interactions, and their interpretation; however, approaches such as cluster analyses have so far provided valuable insights.
- It was not possible to collect blood samples at a standardised time during the day for each participant at each time-point. To account for the diurnal variations in cytokines [41], time of blood collection was recorded for inclusion as a potential confounder when interpreting cytokine levels.

## Supplementary information

**Additional file 1. **Additional Tables.

## Data Availability

All data are held at The University of Queensland, Brisbane, Australia, and handled confidentially in a de-identified format. Currently, only the research team has access to the data. Proposals for collaborative analyses are invited to contact the lead author (Dr David Klyne: d.klyne@uq.edu.au) or principal investigator (Professor Paul Hodges: p.hodges@uq.edu.au).

## Abbreviations

AUDIT: Alcohol Use Disorders Identification Test
BMI: Body mass index
CES-D: Centre for Epidemiological Studies of Depression Scale
CoP: Centre of pressure
CPM: Conditioned pain modulation
CPT: Cold pain threshold
CRP: C-reactive protein
CS: Conditioning stimulus
ELISA: Enzyme-linked immunosorbent assays
EMG: Electromyography
FABQ: Fear-Avoidance Beliefs Questionnaire
FU: Follow-up participants
HPT: Heat pain threshold
IPAQ: International Physical Activity Questionnaire
IQR: Interquartile range
JCQ: Job Content Questionnaire
IL-1β: Interleukin-1β
IL-6: Interleukin-6
LBOS: Low-Back Outcome Scale
LBP: Low back pain
NFU: Non-follow-up participants
NRS: Numerical rating scale
PCS: Pain Catastrophizing Scale
PPT: Pressure pain threshold
PSEQ: Pain Self-Efficacy Questionnaire
PSQI: Pittsburgh Sleep Quality Index
RMDQ: Roland Morris Disability Questionnaire
SD: Standard deviation
TNF: Tumor necrosis factor
TS: Test stimulus
