# Supplementary material for: Cohort profile: why do people keep hurting their back?
Source: BMC Res Notes. 2020 Nov 17;13:538. doi: 10.1186/s13104-020-05356-z (PMC7672992; doi:10.1186/s13104-020-05356-z)
Supplement: Supplementary file 1 — Additional file 1. Additional Tables. [file 13104_2020_5356_MOESM1_ESM.docx]

**Table S1.** Data collection schedule.

|  | **Time-point** | | | | | | | | | | | | | | | | | | | | | | | | |
| --- | --- | --- | --- | --- | --- | --- | --- | --- | --- | --- | --- | --- | --- | --- | --- | --- | --- | --- | --- | --- | --- | --- | --- | --- | --- |
| **Measure** | **0M** |  |  |  |  |  | **3M** |  |  |  |  |  | **6M** |  |  |  |  |  | **9M** |  |  |  |  |  | **12M** |
| **Demographic, health & function:** |  |  |  |  |  |  |  |  |  |  |  |  |  |  |  |  |  |  |  |  |  |  |  |  |  |
| Age, height, sex, BMI, co-morbidities & previous LBP | X |  |  |  |  |  | X |  |  |  |  |  | X |  |  |  |  |  | X |  |  |  |  |  | X |
| Health care and medication usage | X |  |  |  |  |  | X |  |  |  |  |  | X |  |  |  |  |  | X |  |  |  |  |  | X |
| Pain and function (LBOS) | X |  |  |  |  |  | X |  |  |  |  |  | X |  |  |  |  |  | X |  |  |  |  |  | X |
| **Psychological:** |  |  |  |  |  |  |  |  |  |  |  |  |  |  |  |  |  |  |  |  |  |  |  |  |  |
| Depressive symptoms (CES-D) | X |  |  |  |  |  | X |  |  |  |  |  | X |  |  |  |  |  | X |  |  |  |  |  | X |
| Pain catastrophizing (PCS) | X |  |  |  |  |  | X |  |  |  |  |  | X |  |  |  |  |  | X |  |  |  |  |  | X |
| Fearful/avoidant behaviours attributed to LBP (FABQ) | X |  |  |  |  |  | X |  |  |  |  |  | X |  |  |  |  |  | X |  |  |  |  |  | X |
| Pain self-efficacy (PSEQ) | X |  |  |  |  |  | X |  |  |  |  |  | X |  |  |  |  |  | X |  |  |  |  |  | X |
| **Social:** |  |  |  |  |  |  |  |  |  |  |  |  |  |  |  |  |  |  |  |  |  |  |  |  |  |
| Marital status | X |  |  |  |  |  | X |  |  |  |  |  | X |  |  |  |  |  | X |  |  |  |  |  | X |
| Education level | X |  |  |  |  |  | X |  |  |  |  |  | X |  |  |  |  |  | X |  |  |  |  |  | X |
| Employment status | X |  |  |  |  |  | X |  |  |  |  |  | X |  |  |  |  |  | X |  |  |  |  |  | X |
| Type of work | X |  |  |  |  |  | X |  |  |  |  |  | X |  |  |  |  |  | X |  |  |  |  |  | X |
| Job satisfaction (NRS) | X |  |  |  |  |  | X |  |  |  |  |  | X |  |  |  |  |  | X |  |  |  |  |  | X |
| Job stress (JCQ) | X |  |  |  |  |  | X |  |  |  |  |  | X |  |  |  |  |  | X |  |  |  |  |  | X |
| Sick days over last 12 months | X |  |  |  |  |  | X |  |  |  |  |  | X |  |  |  |  |  | X |  |  |  |  |  | X |
| Reason(s) for not working | X |  |  |  |  |  | X |  |  |  |  |  | X |  |  |  |  |  | X |  |  |  |  |  | X |
| Sickness benefits | X |  |  |  |  |  | X |  |  |  |  |  | X |  |  |  |  |  | X |  |  |  |  |  | X |
| Impending compensation associated with LBP | X |  |  |  |  |  | X |  |  |  |  |  | X |  |  |  |  |  | X |  |  |  |  |  | X |
| **Biological:** |  |  |  |  |  |  |  |  |  |  |  |  |  |  |  |  |  |  |  |  |  |  |  |  |  |
| Systemic inflammation | X |  |  |  |  |  | X |  |  |  |  |  | X |  |  |  |  |  | X |  |  |  |  |  |  |
| Pain processing | X |  |  |  |  |  | X |  |  |  |  |  | X |  |  |  |  |  | X |  |  |  |  |  |  |
| Multifidus muscle morphology | X |  |  |  |  |  | X |  |  |  |  |  | X |  |  |  |  |  | X |  |  |  |  |  |  |
| Trunk muscle coordination | X |  |  |  |  |  | X |  |  |  |  |  | X |  |  |  |  |  | X |  |  |  |  |  |  |
| Trunk mechanical properties | X |  |  |  |  |  | X |  |  |  |  |  | X |  |  |  |  |  | X |  |  |  |  |  |  |
| Trunk postural control | X |  |  |  |  |  | X |  |  |  |  |  | X |  |  |  |  |  | X |  |  |  |  |  |  |
| Standing postural control | X |  |  |  |  |  | X |  |  |  |  |  | X |  |  |  |  |  | X |  |  |  |  |  |  |
| Lumbopelvic motion | X |  |  |  |  |  | X |  |  |  |  |  | X |  |  |  |  |  | X |  |  |  |  |  |  |
| Lumbopelvic control during gait | X |  |  |  |  |  | X |  |  |  |  |  | X |  |  |  |  |  | X |  |  |  |  |  |  |
| **Behavioural:** |  |  |  |  |  |  |  |  |  |  |  |  |  |  |  |  |  |  |  |  |  |  |  |  |  |
| Sleep duration and quality (PSQI) | X |  |  |  |  |  | X |  |  |  |  |  | X |  |  |  |  |  | X |  |  |  |  |  | X |
| Physical activity (IPAQ) | X |  |  |  |  |  | X |  |  |  |  |  | X |  |  |  |  |  | X |  |  |  |  |  | X |
| Alcohol use (AUDIT) | X |  |  |  |  |  | X |  |  |  |  |  | X |  |  |  |  |  | X |  |  |  |  |  | X |
| Past/current smoking status | X |  |  |  |  |  | X |  |  |  |  |  | X |  |  |  |  |  | X |  |  |  |  |  | X |
| **Fortnightly:** |  |  |  |  |  |  |  |  |  |  |  |  |  |  |  |  |  |  |  |  |  |  |  |  |  |
| Pain (NRS) | X | X | X | X | X | X | X | X | X | X | X | X | X | X | X | X | X | X | X | X | X | X | X | X | X |
| Disability (RMDQ) | X | X | X | X | X | X | X | X | X | X | X | X | X | X | X | X | X | X | X | X | X | X | X | X | X |
| **12-month trajectory data** |  |  |  |  |  |  |  |  |  |  |  |  |  |  |  |  |  |  |  |  |  |  |  |  | X |

Data were collected for control participants up until 3 months (shaded columns) and for low back pain participants up until 12 months. M – month; BMI – body mass index; LBOS – Low-Back Outcome Scale; CES-D – Centre for Epidemiological Studies of Depression Scale; PCS – Pain catastrophizing Scale; FABQ – Fear-Avoidance Beliefs Questionnaire; PSEQ – Pain Self-Efficacy Questionnaire; NRS – numerical rating scale; JCQ – Job Content Questionnaire; PSQI – Pittsburgh Sleep Quality Index; IPAQ – International Physical Activity Questionnaire; AUDIT – Alcohol Use Disorders Identification Test; RMDQ – Roland Morris Disability Questionnaire.

**Table S2.** Number of low back pain participants classified as either “unrecovered”, “partially recovered” or fully “recovered” at 3, 6, 9 and 12 months.

| **Classification** |  | **3 months** |  | **6 months** |  | **9 months** |  | **12 months** |
| --- | --- | --- | --- | --- | --- | --- | --- | --- |
| Unrecovered |  | 13 |  | 15 |  | 9 |  | 12 |
| Partially recovered |  | 96 |  | 66 |  | 65 |  | 69 |
| Recovered |  | 11 |  | 16 |  | 11 |  | 13 |

For some analyses that have been conducted to date, LBP participants have been classified based on their pain (NRS) and disability (RMDQ) status as either “unrecovered”, “partially recovered” or “recovered” at follow-up after their initial assessment session (i.e., baseline). Participants were classified as: (1) *unrecovered* if they had an increase or no change in pain and disability from baseline, or a pain score of ≥7 of 10 (corresponding with severe pain [1]), (2) *partially recovered* if their pain and/or disability had decreased from baseline, but was unresolved, or (3) *recovered* if they had no pain and disability at the corresponding follow-up time-point. Pain and disability status at each 3-month time-point was calculated by averaging the available data from the final three fortnightly pain NRS and RMDQ scores of the last month prior to that time-point (e.g., 6 months: weeks 20, 22, 24). Participants were classified if pain and disability data were available for at least one of the three fortnightly time-points, even if they did not follow-up for their respective 3-monthly assessment session. Alternative methods for classification of outcome could be applied to the data for future analyses.

**Table S3.** LBP participant inclusion and exclusion criteria.

| **Inclusion criteria** | **Exclusion criteria** |
| --- | --- |
| Ability to understand, speak and read English, and give informed consent | Refusal to participate |
| Within 2 weeks of onset of an acute episode of acute LBP that; | <18 years old  >50 years old (to exclude undiagnosed cardiovascular disease and cancer which would influence inflammatory markers) |
| Occurred between the gluteal fold and T12 | Known or suspected serious spinal pathology (e.g., fracture, metastasis, inflammatory/infective spinal disease, cauda equina syndrome, neurological disorders or symptoms other than leg pain attributed to sciatica) |
| Lasted for >24 hours and remained present at time of study  commencement | Major pain/injury in other body regions in the previous 12 months |
| Caused functional limitation | Other major diseases/disorders (e.g., chronic renal/endocrine disorders) |
| Caused participant to seek/seriously consider health intervention | To control factors that might influence systemic inflammation, participants were also excluded if they were using corticosteroids, non-steroidal anti-rheumatic drugs, or anti-cytokine therapy. Participants could use pain medications that do not affect cytokines and, if required, non-steroidal anti-inflammatory medication provided it ceased 5 days prior to the testing session. Medication was recorded for inclusion as a co-variate. |
| Followed a period of at least 1 month without pain | Exercise at an intensity considered “moderate to high-level” within 24 hours before blood collection |
| Average level of pain (NRS) ≥1 during the week prior to study commencement* | Average level of pain (NRS) <1 during the week prior to study commencement* |
| Average level of disability (RMDQ) ≥1 during the week prior to study commencement* | Average level of disability (RMDQ) <1 during the week prior to study commencement* |

*Confirmed using data from the baseline questionnaire that was completed within 24 hours of the laboratory-based testing session.

**Table S4.** Baseline characteristics of participants with and without LBP.

|  | **Summary statistics** | | |  |
| --- | --- | --- | --- | --- |
| **Characteristic** | **LBP** (N=133) |  | **Control** (N=74) | **P-value** |
| **Demographic, health & function** |  |  |  |  |
| Age (years)^‡^ | 27 (22-34) |  | 25 (21-30) | 0.126 |
| Sex (% female) | 51.9 |  | 60.8 | 0.216 |
| Height (m)^†*^ | 1.73 (0.09) |  | 1.69 (0.11) | **0.028** |
| Weight (kg)^‡*^ | 73 (62-83) |  | 63 (54-73) | **<0.001** |
| BMI (kg/m^2^)^‡^ | 24.0 (21.4-26.9) |  | 22.1 (19.9-24.0) | **<0.001** |
| Comorbidity (yes, %) | 45.1 |  | 28.4 | **0.018** |
| Previous LBP (yes, %) | 91.7 |  | 31.1 | **<0.001** |
| Healthcare utilization (yes, %) | 19.7 |  | NA | NA |
| Medication utilization (yes, %) | 22.4 |  | NA | NA |
| Function (LBOS)^†^ | 46.9 (11.3) |  | NA | NA |
| **Outcome** |  |  |  |  |
| Pain (NRS)^‡^ | 5 (4-7) |  | NA | NA |
| Disability (RMDQ)^‡^ | 6 (4-9) |  | NA | NA |
| **Psychological** |  |  |  |  |
| Depressive symptoms (CES-D)^‡^ | 11.5 (8-19) |  | 7 (4-12) | **<0.001** |
| Pain catastrophizing (PCS)^‡^ | 10.5 (6.5-20) |  | 2.5 (0-12) | **<0.001** |
| Fear avoid.-work (FABQ-W)^‡^ | 12 (3-18) |  | NA | NA |
| Fear avoid.-activity (FABQ-PA)^‡^ | 15 (11-19) |  | NA | NA |
| Pain self-efficacy (PSEQ)^‡^ | 47 (36-51) |  | NA | NA |
| **Social** |  |  |  |  |
| Marital status (not married/cohabitating, %) | 63.6 |  | 71.6 | 0.244 |
| Edu. level (secondary school/below, %) | 25.0 |  | 31.1 | 0.347 |
| Empl. status (unemployed, %) | 22.7 |  | 33.8 | 0.085 |
| Job satisfaction (NRS)^‡^ | 4 (3.5-5) |  | 4 (3-5) | 0.703 |
| Job skill discretion (JCQ)^‡^ | 36 (32-40) |  | 36 (28-42) | 0.640 |
| Job decision-making authority (JCQ)^‡^ | 40 (32-44) |  | 36 (28-40) | 0.104 |
| Job demands (JCQ)^†^ | 32.4 (6.5) |  | 29.6 (5.9) | **0.0115** |
| Job decision latitude (JCQ)^‡^ | 74 (66-84) |  | 70 (62-80) | 0.211 |
| Co-worker support (JCQ)^‡^ | 12 (12-14) |  | 13 (12-15) | 0.102 |
| Supervisor support (JCQ)^‡^ | 12 (11-14) |  | 12 (12-16) | 0.149 |
| Job insecurity (JCQ)^‡∆^ | 5 (4-7) |  | 5 (4-7) | 0.914 |
| Sick days over last 12 months^‡*^ | 2 (0-4 |  | 0 (0-3) | **0.021** |
| Sickness benefits for LBP (yes, %) | 1.9 |  | NA | NA |
| Impending compensation (yes, %) | 9.3 |  | NA | NA |
| **Behavioural** |  |  |  |  |
| Sleep quality (PSQI)^‡^ | 9 (7-11) |  | 5 (4-7) | **<0.001** |
| Alcohol use/related problems (AUDIT)^‡^ | 3 (2-6) |  | 3 (1-5) | 0.121 |
| Previous/current smoker (yes, %) | 36.4 |  | 21.9 | **0.033** |
| Current smoker (yes, %) | 7.6 |  | 2.7 | 0.158 |
| Vig. phys. activity days/week (IPAQ)^‡*^ | 2 (0-3) |  | 2 (0-4) | **0.034** |
| Vig. phys. activity time/day (min, IPAQ)^‡*^ | 20 (0-60) |  | 34 (0-60) | 0.054 |
| Mod. phys. activity days/week (IPAQ)^‡*^ | 2 (0-4) |  | 3 (0-5) | 0.419 |
| Mod. phys. activity time/day (min, IPAQ)^‡*^ | 30 (0-60) |  | 43 (0-60) | 0.256 |
| Days/week walking for ≥10 min (IPAQ)^‡*^ | 6 (4-7) |  | 7 (4-7 | 0.428 |
| Walking time/day (min, IPAQ)^‡*^ | 30 (20-60) |  | 30 (20-60) | 0.657 |
| Sitting time/day (min, IPAQ)^‡*^ | 420 (240-540) |  | 420 (300-585) | 0.619 |

Variable (characteristic) summary statistics (mean [SD]^†^, median [IQR]^‡^ or percentage) compared between low back pain and control participants using *t* tests (continuous data, normally distributed), Mann-Whitney *U* tests (continuous data, not normally distributed) or Chi squared tests (categorical data). Edu. – education; Empl. – employment; Vig. – vigorous; Mod – moderate; min – minute. Refer to Tables 1 and S1 for other abbreviations.

*If a participant provided a range of values in response to a question (e.g., 30-60 minutes), the average was calculated for analysis purposes.

^∆^Participants who answered “other” to *question 25* of the Job Content Questionnaire were removed prior to analysing the “job insecurity” scale.

**Table S5.** Number of baseline and follow-up participants that provided valid data for each of the 3-monthly questionnaire-based measures at 3, 6, 9 and 12 months.

| **Measure** | **Baseline (N=133)** |  | **3 months (N=98)** |  | **6 months (N=89)** |  | **9 months (N=84)** |  | **12 months (N=92)** | |
| --- | --- | --- | --- | --- | --- | --- | --- | --- | --- | --- |
| **Demographic, health & function** |  |  |  |  |  |  |  |  |  |  |
| Age | 133 |  | NA |  | NA |  | NA |  | NA |  |
| Sex | 133 |  | NA |  | NA |  | NA |  | NA |  |
| BMI | 133 |  | 72 |  | 61 |  | 51 |  | 65 |  |
| Comorbidity (number/type) | 132 |  | 71 |  | 59 |  | 46 |  | 65 |  |
| LBP history | 133 |  | NA |  | NA |  | NA |  | NA |  |
| Healthcare utilization (freq./type) | 132 |  | 71 |  | 58 |  | 46 |  | 63 |  |
| Medication utilization (freq./type) | 107 |  | 61 |  | 50 |  | 44 |  | 61 |  |
| Function (LBOS) | 132 |  | 71 |  | 58 |  | 46 |  | 62 |  |
| **Outcome** |  |  |  |  |  |  |  |  |  |  |
| Pain (NRS) | 133 |  | 95 |  | 84 |  | 73 |  | 85 |  |
| Disability (RMDQ) | 133 |  | 95 |  | 85 |  | 73 |  | 87 |  |
| **Psychological** |  |  |  |  |  |  |  |  |  |  |
| Depressive symptoms (CES-D) | 132 |  | 70 |  | 58 |  | 46 |  | 62 |  |
| Pain catastrophizing (PCS) | 132 |  | 71 |  | 60 |  | 46 |  | 62 |  |
| Fear avoid.-work (FABQ-W) | 132 |  | 71 |  | 57 |  | 46 |  | 62 |  |
| Fear avoid.-activity (FABQ-PA) | 132 |  | 71 |  | 57 |  | 46 |  | 62 |  |
| Pain self-efficacy (PSEQ) | 107 |  | 61 |  | 50 |  | 44 |  | 60 |  |
| **Social** |  |  |  |  |  |  |  |  |  |  |
| Marital status | 132 |  | 71 |  | 58 |  | 46 |  | 62 |  |
| Education level | 132 |  | 71 |  | 58 |  | 46 |  | 63 |  |
| Employment status | 132 |  | 71 |  | 58 |  | 46 |  | 63 |  |
| Job Content Questionnaire (JCQ) (NRS) | 108 |  | 61 |  | 52 |  | 41 |  | 58 |  |
| Job skill discretion (JCQ) | 103 |  | 59 |  | 52 |  | 42 |  | 57 |  |
| Job decision-making authority (JCQ) | 104 |  | 59 |  | 52 |  | 43 |  | 57 |  |
| Job demands (JCQ) | 105 |  | 59 |  | 52 |  | 42 |  | 57 |  |
| Job decision latitude (JCQ) | 102 |  | 58 |  | 52 |  | 42 |  | 57 |  |
| Co-worker support (JCQ) | 105 |  | 60 |  | 52 |  | 43 |  | 57 |  |
| Supervisor support (JCQ) | 82 |  | 52 |  | 45 |  | 42 |  | 57 |  |
| Job insecurity (JCQ)^∆^ | 99 |  | 54 |  | 51 |  | 41 |  | 57 |  |
| Sick days over last 12 months | 98 |  | 54 |  | 41 |  | 35 |  | 49 |  |
| Sickness benefits for LBP | 107 |  | 61 |  | 50 |  | 44 |  | 63 |  |
| Impending compensation | 107 |  | 61 |  | 50 |  | 44 |  | 63 |  |
| **Behavioural** |  |  |  |  |  |  |  |  |  |  |
| Sleep quality (PSQI) | 113 |  | 70 |  | 57 |  | 47 |  | 62 |  |
| Alcohol use/related problems (AUDIT) | 132 |  | 71 |  | 58 |  | 46 |  | 65 |  |
| Smoking history | 132 |  | 72 |  | 57 |  | 45 |  | 65 |  |
| Physical activity (IPAQ) | 132 |  | 72 |  | 58 |  | 47 |  | 65 |  |

Refer to Tables 1 and S1 for abbreviations. ^∆^Participants who answered “other” to *question 25* of the Job Content Questionnaire were removed prior to analysing the “job insecurity” scale.

**References**

1. Boonstra AM, Preuper HRS, Balk GA, Stewart RE. Cut-off points for mild, moderate, and severe pain on the visual analogue scale for pain in patients with chronic musculoskeletal pain. Pain. 2014;155(12):2545-50. doi: 10.1016/j.pain.2014.09.014. PubMed PMID: WOS:000345414700016.
